# Please understand when I cry out in pain: women's accounts of maternity services during labour and delivery in Ghana

**DOI:** 10.1186/1471-2458-5-140

**Published:** 2005-12-22

**Authors:** Lucia D'Ambruoso, Mercy Abbey, Julia Hussein

**Affiliations:** 1 IMMPACT, University of Aberdeen, Aberdeen, UK; 2 Health Research Unit, Ghana Health Service, Accra, Ghana

## Abstract

**Background:**

This study was undertaken to investigate women's accounts of interactions with health care providers during labour and delivery and to assess the implications for acceptability and utilisation of maternity services in Ghana.

**Methods:**

Twenty-one individual in-depth interviews and two focus group discussions were conducted with women of reproductive age who had delivered in the past five years in the Greater Accra Region. The study investigated women's perceptions and experiences of care in terms of factors that influenced place of delivery, satisfaction with services, expectations of care and whether they would recommend services.

**Results:**

One component of care which appeared to be of great importance to women was staff attitudes. This factor had considerable influence on acceptability and utilisation of services. Otherwise, a successful labour outcome and non-medical factors such as cost, perceived quality of care and proximity of services were important. Our findings indicate that women expect humane, professional and courteous treatment from health professionals and a reasonable standard of physical environment. Women will consciously change their place of delivery and recommendations to others if they experience degrading and unacceptable behaviour.

**Conclusion:**

The findings suggest that inter-personal aspects of care are key to women's expectations, which in turn govern satisfaction. Service improvements which address this aspect of care are likely to have an impact on health seeking behaviour and utilisation. Our findings suggest that user-views are important and warrant further investigation. The views of providers should also be investigated to identify channels by which service improvements, taking into account women's views, could be operationalised. We also recommend that interventions to improve delivery care should not only be directed to the health professional, but also to general health system improvements.

## Background

Recent figures indicate that globally, 529,000 women die in pregnancy and childbirth each year [1]. The detrimental effect of maternal death on household income, household productivity, and household disintegration has been widely described [2-4]. In addition, maternal deaths cause 1 million children to become motherless annually [5,6]. There are estimates that for every maternal death, another 20 women will develop some form of life-long morbidity related to pregnancy and/or childbirth [6]. This is a considerable burden of disability and disease. It has also been demonstrated that 99% of the world's maternal deaths occur in developing countries [1,2,7,8]. Concern for the significant mortality and morbidity associated with pregnancy and childbirth is therefore prominent on global health agendas [9].

Global advocacy groups describe maternal mortality as "avoidable" and "preventable" [6,10]. This might be because over 70% of maternal deaths are due to five major complications (haemorrhage, sepsis, unsafe abortion, eclampsia and obstructed labour [11]) and the clinical means to prevent either deaths arising from these complications, or the complications themselves, are well known. However, health programmes and initiatives that target this health outcome have not always experienced a significant degree of success [12]. Recent evidence suggests that part of the problem with not achieving reductions in maternal mortality might be due to more than the efficacy of interventions. Studies have demonstrated that health systems factors, service delivery and the inter-personal aspects of care also play an important part [2,11,13]. Badly delivered and poor quality health care services have been seen to compromise "access to services, compliance and effectiveness" [14-16].

Lack of success may also be related to issues of access. Maternal deaths could be prevented if women were able to access and utilise good quality services, especially when complications arise [17]. However, in reality, most women experience serious barriers to accessing services or even if they do reach them, the services themselves are often of insufficient quality or effectiveness. Interventions that generate demand for care are likely to, for example, increase the chances that women will gain this access to services. In recent years, system or sector-wide strategies focussed on skilled attendance have aimed to generate demand as well as augment supply. Skilled attendance is now widely advocated as the single most crucial intervention to reduce mortality owing to pregnancy and childbirth [11,18,19]. The rationale is based upon the potential for trained health workers to manage cases appropriately and prevent complications. The components of skilled attendance are well documented and include the health professional within an 'enabling environment' which comprises a functioning health system including effective transportation, drugs, equipment and supplies [11,20-22]. The enabling environment also requires effective links between services and the community [18].

Considering the perspective of the service-user has been seen to increase the acceptability of services [23,24]. However, the influence of inter-personal aspects of care, as a component of quality, has not been investigated as extensively as other, more clinical, elements. Patient satisfaction is a related concept as it aims to determine "individual perceptions of the quality of health care delivered" [25]. Although studies of patient satisfaction, which often focus on patient-provider interactions, have increased in numbers in industrialised nations in recent years [25], considerable difficulties surround the conceptualisation and measurement of satisfaction. In addition, respondents have been seen to report unrealistically high levels of satisfaction which are likely to be prone to courtesy or gratitude biases [24-27].

Despite these difficulties, service planning which *does *recognise and address the influence of the patient-provider interaction has been seen to be an effective means by which to improve the quality of services [2,13,28]. Such service-improvements have been shown to influence utilisation and compliance and have resulted in a "larger, more committed clientele" [23]. In the longer term, this translates into better quality of care and ultimately, improvements in health outcomes. This study was undertaken with the objective of investigating women's accounts of interactions in delivery care and to assess their implications for acceptability and utilisation of maternity health care services in Ghana.

Ghana is situated in West Africa with a population of approximately 19 million [29]. Ghana is classified as a low-income country and has a gross national product (GNP) per capita of $270 [30]. In 2003, almost a third of women aged 15–49 had no education. 12% of all pregnancies do not result in a live birth and less than half of all births are attended by a qualified health professional [31]. Over 70% of babies born are not weighed at birth and nearly 50% of non-institutional deliveries receive no postnatal care [31]. Although this study was concerned with deliveries attended by health professionals, it should be noted that within the Ghanaian context, and particularly in rural Ghana, utilisation of informal health care services such as traditional birth attendants (TBAs) is common, and TBAs attend up to half of all births [31,32].

This study was part of a research initiative called SAFE (Skilled Attendance for Everyone) which aimed to improve the knowledge base on skilled attendance at delivery in developing countries [19,22]. The accounts of labour and delivery discussed in this paper arose out of a wider study, published elsewhere [33] investigating how women identify their delivery attendants. During the course of the wider survey, women spontaneously shared their views on delivery experiences and factors were identified that warranted further investigation, prompting us to conduct this exploratory study. Our results are targeted at service providers, managers and planners as evidence suggestive of factors related to acceptability and utilisation. Possible routes for further investigation are proposed as are some recommendations for practice.

## Methods

Focus group discussions (FGDs) and individual in-depth interviews were the techniques employed. Qualitative techniques were appropriate since women were talking about sensitive, personal issues. Although the original intention was to conduct FGDs, it was quickly established that women were reluctant to talk about their experiences using this method. Since the experience of labour is unique, intimate and highly personal for every woman, it was difficult for participants to freely share the details of their particular experiences especially in a group discussion. It was concluded that the study should be continued using in-depth interviewing. With this approach, women provided detailed accounts of their experiences.

Changing the methodology during the course of the study from FGDs to in-depth interviews was a reactive way to respond to the unsatisfactory results of the FGDs and maximise upon the opportunity to collect meaningful data. The results of the FGDs were used to develop the in-depth interview guide and were analysed with the results of the interviews. There were some potential problems with this approach. Analysing the FGD and interview data together ran the risk of 'diluting' the importance of factors identified in the interviews that may not have been as pertinent in the FGDs. However this risk was deemed to be favourable to the alternative of loss of key issues that arose during the FGDs. In addition, since women were asked in both the interviews and FGDs to consider the same issues, the data was sufficiently consistent. However, during the course of the analysis, we took care to maintain this consideration in terms of the conclusions we arrived at.

The theoretical basis to the methodology originates in the constructivist paradigm towards enquiry. This paradigm sets out that human beings individually and collectively interpret or construct the social and psychological world in specific social, linguistic and historical contexts. The theory asserts that knowledge and truth are created through the perspective of human beings, and are not discovered [34]. Women's perception of their care during delivery is, thus, best described and interpreted by themselves. The interviews and FGDs used semi-structured guides and included open-ended questions (see Table 1). Open-ended questions encouraged women to describe their experiences in their own terms and using their own language. This allowed them to freely recount factors that were of importance to them without being influenced by the line of questioning.

**Table 1:** Topics of semi-structured interviews and FGDs

- Place of delivery
• awareness of place of delivery
• availability of place of delivery
• actual place of delivery
• reasons for choice
- Satisfaction with services
- Expectations of care
- Recommendations of services to other women.

A sample size was not calculated as this was not appropriate for the qualitative method. Women attending antenatal and child welfare clinics and who had delivered with a health professional were included in the study. The opportunity to conduct our study arose during the course of another piece of research [33] that recruited women who had delivered with a health professional in the last five years. The sample of women included in the study we report here was thus opportunistic. The five year recall period was originally chosen as women's recollection of obstetric events even over long periods of time has been demonstrated as accurate and experiences are recalled in great detail [35-39]. However, this may apply to more tangible events (e.g. number of births or deliveries) and subjective elements (such as feelings and satisfaction) may change over time. While acknowledging the potential for recall bias, we were able to draw upon the combined perceptions of women's birth experiences over the five year period.

Two FGDs and twenty-one individual in-depth interviews were conducted in suburbs of Accra, areas which were semi-urban in nature. Data were collected between April and September 2001. Approval for the study was obtained through the Ministry of Health in Ghana. Women participating in the study gave verbal consent.

Interviews and FGDs were conducted in various locations – health facilities and women's homes, the place of the interview being chosen by the respondent. When it did not inconvenience the woman to be interviewed immediately, the interview was conducted in a quiet area in or near the facility. In other cases, women requested that the interviewer visit them at home, and so the interview was conducted there at a later date. Conducting an interview or FGD in a health facility location might have biased some respondents to give accounts of care that may have been more positive, and less reflective of their true perception. However, it was felt that it was more appropriate, and that this courtesy bias could be minimised, by offering women a choice of interview setting.

The main researcher was trained in qualitative methodology and conducted both the interviews and the FGDs. A tape recorder was used to record the sessions and a transcript of the discussions was made by an independent person. The transcripts were translated into English, reviewed and coded to identify pertinent themes. The approach to both the data collection and analysis was exploratory and did not pre-suppose any relationship or significance of the factors which were identified. The analysis was thematic to draw out the main themes and contradictions. A theme needed to recur in a (non-statistically) significant proportion of the women, to indicate that it was an important issue. When emergent themes had recurred sufficiently, it was appropriate to conclude the interviews as no new themes were likely to emerge, i.e. "thematic saturation" had occurred. The results are discussed according to the prominent themes in the following sections. Some findings are presented verbatim of the in-depth interviews and FGDs, others are summarised. Our approach has been not to assign quotes to specific individuals because the small numbers of interviews allows us to represent the opinions from the entire range of women involved.

## Results

### Characteristics of women

The women included in the study were aged between 18 and 38 years. Parity was from one to four children. The outcome of pregnancy in all cases was a live birth. Most women had some basic education with the exception of a few who had never been to school. Most were married and engaged in various trades such as hairdressing, dressmaking and petty trading; few were unemployed.

### Women's accounts of care

The FGDs and interviews were arranged around four topics (see Table 1). Recurring themes that spontaneously emerged from women's expressed opinions reflected components of care that were of importance and which influenced their satisfaction with services.

#### Place of delivery

Generally women were aware of the existence of various facilities for delivery within the area of their residence. These facilities were both private and public with a range of sophistication in terms of emergency obstetric care. They included private and public maternity homes with basic obstetric care but without operating theatre facilities and a resident obstetrician, to facilities with specialist obstetric care.

The majority of women delivered in public health facilities. More women delivered in facilities with specialist obstetric care than in public facilities with basic obstetric care. The rest delivered in private facilities. Many women who delivered in specialist obstetric facilities had been referred there during antenatal attendance due to their risk status or because of availability of specialist care.

"They told me I had waited too long. They said at age 30 I was too old to have my first child and therefore asked me to go to (hospital A)."

"I delivered at the (hospital B) because I was referred there after staying too long in labour in a maternity home."

"I delivered at (health facility A) because this is my first child. If you delivered anywhere else and you develop a complication they would refer you there eventually and so I decided to go there as a first choice."

When asked what factors would affect the choice of delivery facility, most of the interviewees specified staff attitudes. In terms of medical outcomes, a successful outcome of labour and delivery for mother and child was also of high importance:

"...after-all what is important is the goal of the activity: to come out as a live and healthy mother with a healthy baby."

Poor outcome of previous pregnancy, such as fresh stillbirth, and perceptions of poor quality of care deterred women from choosing certain facilities for delivery. This was especially clear where mothers had the perception that the outcome could have been otherwise but for the care she received:

"You see some of the mothers who die and lose their babies are as a result of the action of the nurses. If it had not been for the other nurse, my baby could have dropped to the floor and anything could have happened."

"I delivered at (health facility B) because it is cheaper. If I had money I would have delivered somewhere else because my previous experience at (health facility B) I lost my baby."

Other factors that influenced choice of facility were cost of services, access and recommendation by family and friends. Otherwise, other previous experience, administrative arrangements (i.e. an arrangement between a company and a health facility for the provision of care for its employees and their families), the general environment of the facility (i.e. level of noise or orderliness, sanitation and neighbourhood), the availability of a known person or family member in that facility, proximity of a facility to family members who could assist in caring for mother and child, confidentiality and privacy influenced choices:

"I have no relatives here to take care of me so I went to deliver where my relatives are."

"My husband asked me to go that facility because the cost will be moderate."

"...it also depends on your husband's "pocket."

"...it is the common facility for delivery for people in this community. It is also near and it is our (hospital A)."

The availability of friends or family members for post-delivery assistance was important to women. Women indicated that they travel long distances to deliver in facilities that are close to friends or relatives.

#### Satisfaction

In describing their satisfaction with services, the women interviewed talked about delivery experiences with their last child and previous deliveries (where the woman had previous deliveries). Women accounts depicted both positive and negative encounters with staff.

"I like the place I went to. They helped me deliver, gave me a nice place to sleep and gave me advice on how to feed the baby."

"The people there are very good. They prayed with me and taught me how to care for the baby."

"The services were not so good, the attendant ... refused when I needed to hold her while I was in pain she said it won't change anything...even when I asked the ward assistant for water she brought me chilled water, when I said I preferred tap water, she became angry."

"One nurse refused to make a cup of tea for me. When I requested for it she insisted that I make it for myself although she knew I was in pain."

"... the attendants were angry with me when I was in labour. They were impatient with me."

Women who had appalling treatment expressed indignation. For some women, reliving their experiences caused much pain and they cried on recollection.

"The nurse put my finger into my vagina and asked what I could feel. I said it was the baby's head and I asked her whether I should push. She retorted 'What are you lying there for?"'

"... nurse was so nasty and put fear in me and threatened that they would take me to the theatre if I dared push again. She said because of the pushing I had soiled my pad and so she ordered that I should go and dispose of it myself. In fact this was difficult, but I had to crawl to the disposal bin. When she came and realized the baby was out she asked me why I had not told her the baby was about to come. But I did not know. It is her job and should have known that the baby was coming. She was the one who had listened to the baby and had attended to me. How could I have known that the baby was about to come out?"

Other aspects of services with which women were not satisfied included high cost and evidence of poor quality; crowding babies on the same bed; inadequate numbers of nurses to attend to women in labour; no local anaesthetic for episiotomy suturing; unduly waiting before weighing the baby; and asking mothers to vacate beds regardless of time and inconvenience.

#### Expectations

Expectations were generally governed by experience (the woman's own and/or other's) which influenced their future expectations. All women expressed a desire to have staff with a positive attitude. Some mothers described the positive staff attitudes they would expect. These included giving reassurance, encouragement and politeness, provision of mosquito nets, patience and tolerance. When asked about expectations, accounts of experiences were given to illustrate care which they would (or would not) expect:

"The main person who assisted me was the nurse who asked me to be patient, kept coming to see how I was faring. She was the one who treated me kindly, performed the episiotomy and sutured it. When I bled on the floor, she cleaned. She received the baby and the placenta. She cut the cord and made sure the placenta was out."

Women expected attending midwives to provide guidance and counselling. However, the accounts indicated that providers expected women to know what to do at various stages during labour and delivery and that their lack of knowledge attracted reprimand from some attending nurses and midwives. Other descriptions of poor staff attitude included, rudeness, undeserved or inappropriate reprimand, shouting at women in labour, lack of empathy, refusal to assist, refusal to allow woman in labour to touch or hold a midwife, threatening patients in labour with poor outcomes if they did not comply with instructions, denying women service and lack of moral support and encouragement to exhausted women in labour:

"The midwives I met when I arrived were nice to me, but one of those who came to relieve them kept shouting at me and told me to keep quiet while screaming in pain. I'm glad she didn't attend my delivery."

The following excerpts relate to what women would have liked their attendant to do during the labour and delivery.

"I prefer my attendant to reassure me, be tolerant and encourage me through the stages."

"The attendant should be patient with me since it is my first delivery."

"They should be patient and tolerant when I am in pain and shouting. Also when I can't push properly they should be understanding."

"The facility should acquire an ambulance to make it easy to transfer women in cases of emergency."

Women expected humane and courteous treatment during labour and consciously changed their place of delivery if they experienced degrading and unacceptable behaviour.

"I wanted to deliver in (health facility C) but while attending ante natal clinic, a nurse was rude to me so I changed my mind and delivered in another facility."

#### Recommendations of women

When asked if they would recommend the services, women considered the performance of attendants and the services they had received. Recommendations were mainly due to positive attitudes of one or more providers. Likewise, others would not make recommendations because of negative experiences:

"I lost my first baby at a facility ... and that's how come my friend introduced me to a Gynaecologist in different facility where I had my subsequent children."

"I will recommend that facility because the providers were caring... the Nurse asked me to pray and the doctor said what he would do was going to be painful; but he reassured me. They injected, me again (when I said it was painful) to make it bearable."

"I will go there again because even though one of the nurses was unfriendly and impatient, the other was very accommodating and I pray I will meet someone like her anytime I have to go there."

"...she was standing by me and praying.... I will recommend her."

"Compared with my previous delivery attendant this one was very good, polite, patient and reassuring. Therefore I will recommend her."

However, some accounts suggested that women would recommend services despite abusive behaviour in order to have a 'safe' (facility) birth, underlining the importance of a successful birth outcome:

"I will recommend the facility to others because if you have any problem with your delivery, that is where you will be referred to (referral facility). Despite the shouting and unfriendly behaviour I think the outcome to have your baby and be alive is the most important."

Women commended staff, especially those who showed respect and concern for them in labour. They showed clear abhorrence for those who were abusive and in general, were determined to avoid any further contact with them, directly or indirectly and would not recommend their services.

## Discussion

### Study limitations

#### Context

The study examined women's perspectives of delivery experiences with health professionals, so only women who had delivered in facilities were interviewed as home deliveries with health professionals are rare [31]. As stated in the introduction, institutional deliveries are not common in Ghana; only two in five births take place in a health facility and less than half of all births are attended by a qualified health professional [29,31,40]. Furthermore, the study was confined to a semi-urban area where facility deliveries with health professionals are more common. The results of the study therefore, have implications primarily for care related to deliveries that occur in health facilities, attended by health professionals.

#### Respondents

The age range of respondents was 18–38 years. Women of reproductive age (15–49 years) will have been accessing the antenatal and child welfare services where recruitment to the study took place. In addition, women who had had a facility delivery were likely to be younger, low parity women [31]. This may be why the age-range of the respondents fell short of the women of reproductive age-range. The age-range may have given rise to particular perceptions, recollection of care etc, i.e. younger, less experienced patients are likely to have fewer expectations and so be more satisfied with services. By contrast, older patients have been seen to be more satisfied and compliant with care than their younger counterparts [41-44]. As recruitment to the study was voluntary, it is also possible that women who were most unhappy with services might have declined to be interviewed. Other factors such as wealth and education can also be influential on perceptions [41-44]. However, despite the socio-economic and demographic differences between respondents, there was a considerable level of consistency in the factors that were identified as determinants of satisfaction, and consequently access to, utilisation of and compliance with care.

#### Methodology

The study has raised concerns about the appropriateness of the FGD method in capturing information on personal experiences. In the group discussions, dominant participants could set the tone about how easy their delivery had been. The outcome of the pregnancy in all cases was a live birth. This may have been sufficient to over-shadow any inconveniences experienced during labour for young, mostly primiparous, mothers. This was coupled with very little with which to compare their first experience. We therefore had to alter the method to individual in-depth interviews to provide privacy, confidentiality and non-competitiveness. Participants were more relaxed and provided detailed insight into their experiences with this method. It was concluded that the interview technique was more sensitive and effective than the focus group.

Although the qualitative approach taken was appropriate and yielded data which had more depth than would have been collected by a quantitative survey, and despite the internal consistency of the results, the external validity of the findings is low. Although the women involved in the study were broadly comparable with those of the wider population in Ghana, in terms of education and employment [31], the findings have relevance only to those groups of women involved in the study. However, studies investigating patient satisfaction and inter-personal aspects of care have been seen to be most successful and accurate when they are applied to specific interests and investigated by way of local surveys [25].

#### Other potential biases

Respondents' accounts of subjective events around pregnancy and childbirth may have been prone to recall bias, particularly with a recall period of up to five years. There also may have been courtesy bias – women may have felt that expressing negative feelings about their care may have implications for future care. There was an attempt to minimise the latter by allowing the women to choose the location of the interview. With regard to the potential for recall bias, studies show that recollection of various factors related to pregnancy and delivery is accurate, even over long periods [35-39]. In addition, a recent study found that dissatisfaction with care in childbirth may even emerge over time as relief or gratitude for a safe delivery subside [45]. The same study recommends that investigations of women's birth experiences are more accurate if conducted some time after delivery. The recall period, therefore, may have helped to address the courtesy bias, although the results should be interpreted with the potential for these biases in mind.

### Theoretical frameworks

We implicitly assumed that the accounts of women's experiences, the acceptability of and satisfaction with services reflected, to some degree, the quality of the services. As such, elements of theoretical frameworks of satisfaction and skilled attendance can be illuminating when interpreting the results.

In a review of satisfaction-theory, Sitzia and Wood infer that expectations govern satisfaction, i.e. the more a service meets with the expectations of a user, the more that user will be satisfied with that service [25]. When we examined expectations we found that women almost exclusively expect positive attitudes from staff. The influence of staff attitudes can be seen in the recommendations women made, the choice of delivery facility and their satisfaction with services. It is also noteworthy that Sitzia and Wood dispute high levels of satisfaction equate with high quality care. They infer that high levels of satisfaction commonly reported suggest that "dissatisfaction is only expressed when an extreme negative event occurs" [25], which may be cause for concern in light of our results.

**Figure 1:**
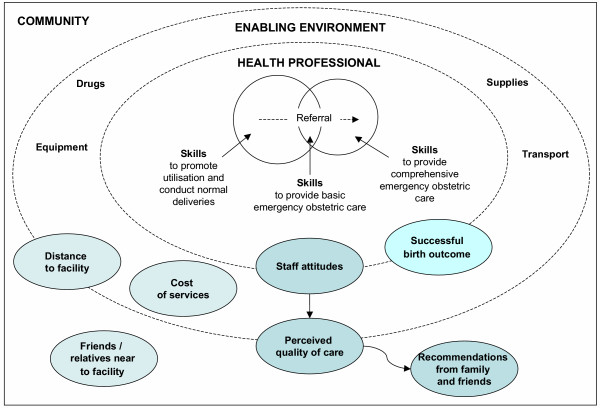
Categorisation of components of care which women perceive as important during delivery to the SAFE framework, with indication of the influence of staff attitudes.

The results also fit within standard theoretical frameworks of skilled care. The SAFE conceptual framework illustrates the key elements of skilled attendance as the skilled attendant and the enabling environment [22]. Figure 1 illustrates the original framework with the components of delivery care referred to by women as important, super-imposed upon the framework in shaded ellipses. This helps to reinforce the important inter-relationships and influences between the professional, the enabling environment and the wider community, particularly since the majority of important factors relate to more than one element of the framework. For example, perceived quality of care is a construct of the community, by virtue of its 'perception'. However, this perception is also governed by factors such as availability of equipment and beds etc, which are attributable to the enabling environment. The lines separating the elements of the framework are dotted to illustrate these inter-relationships.

Our findings bring an additional perspective to the framework – the interpersonal aspects of care, and their influence. The original framework focuses on the health professional in terms of their clinical skills and the clinical aspects of the enabling environment [20-22]. Our results help to emphasise the importance and influence of staff attitudes, which can be linked throughout the conceptualisation from the professional to the enabling environment, affecting perceived quality of care, to the community affecting the recommendations women make and receive. It should also be acknowledged that women are likely to construct their expectations on the basis of the experiences of others, as well as their own. The willingness of women to travel long distances to be close to family and friends also indicates the importance of social, community and familial factors. From this diagram and the Sitzia and Wood discourse on satisfaction [25], we can infer that health professionals have the potential to influence some or all of the elements of skilled care *and *a major determinant of satisfaction – women's expectations, which in turn affects utilisation.

How to feed back the results of the research was discussed with district health officers and a meeting of health professionals in the study area was agreed upon. Although it was not feasible to have all health professionals attend the meeting, it was recognised that the implications for practice from the study were important and further feedback was organised via the meeting attendees to their colleagues. This approach was favourable as it may have been threatening for health professionals to receive this information from researchers and district health officers, and was more conducive to change if fed back from colleagues. At the meeting, many professionals expressed surprise and lack of awareness of these perceptions. Whether providers are even aware of women's needs and expectations as they arrive for delivery is debatable. What might seem to be a normal, rational practice, can have pronounced, detrimental effects on a woman's subsequent health seeking behaviour. In studies from disparate nations such as South Africa, the United States and Brazil, reasons for such prominent negativity from staff towards patients have been suggested to be used to exert control and obedience, command and respect [14,16,23,42]. Other cultural and historical systematic factors have been seen as influences on providers' behaviour in other parts of Africa with financing, training, infrastructure, attitudes and beliefs of social ranking having an impact [16].

### Recommendations

This study adds weight to the increasing recognition that taking into account women's perceptions is crucial to improve the delivery care experiences of women [46-48]. The results of our study, particularly the themes concerning patient-provider relationships, are congruent with those seen in both developed and developing countries – that positive experiences have a profound influence on acceptance, uptake and use of services and affect demand, compliance, uptake and quality of care [23,25,28,43,49-53].

The means to address the factors relating to good delivery care practices are therefore to ensure a high level of awareness amongst practitioners. Sitzia and Wood describe several mechanisms by which patient views might be communicated to the health system from patient-provider discussions, patient advocates, patient comment-boxes in hospitals, patient committees, complaints committees, focus groups, public meetings and surveys [25]. Additionally, training and supervisory interventions that encourage acquisition of interpersonal skills are highly recommended [54]. International organisations such as the World Health Organization (WHO), the International Federation of Gynecology and Obstetrics (FIGO) and the International Confederation of Midwives (ICM) have defined a minimum set of skills for providers [55-57] that go beyond clinical skills to include a wide range of interpersonal and attitudinal skills. These include general care and counselling of women, cultural sensitivity, appropriate communication skills, provision of psychological support and involving women, parents and families in provision of care.

The study was dependant on interviews, women's subjective accounts of the care they received. A broader sense of the issues could be obtained by observing interactions, and including perspectives of women who choose to deliver outside facilities and without a health professional. Provider's accounts of care are an even less examined area [13,28,52,58] although they have been stated as "a crucial component of any attempt to change institutional protocols" [24]. We would therefore recommend that providers' perspectives and motivations are investigated in conjunction with descriptions of user-views, as two parts of a whole, in order to identify effective mechanisms by which needs of the users can be responded to, to ultimately increase quality of care.

The framework we use helps to highlight that interventions to correct the situation should not only be directed toward the health professional. Many of the expectations of women for good quality care lie beyond the capacity of the health professional to provide. The Ghanaian government is committed to improving the quality of their population and reproductive health programmes [31]. There is acknowledgement that general health system improvements addressing the cost of care, availability of human resources, equipment or infrastructure – i.e. providing an "enabling environment" – are key to providing good delivery care. It should also be noted that other factors such as personal needs and social and familial influences may not be possible to improve as part of service provision.

A final recommendation resulting from this study relates to the need for continuing research and documentation of women's perspectives. Even when birth outcomes are successful some of the accounts of care depict serious neglect and abuse. There is a need to share common responsibility for ensuring that research, policy and programming address these serious malpractices, sub-standard care and lack of "woman-friendliness" in maternity services.

## Conclusion

Our findings suggest that health professionals' attitudes towards patients is a critical element of care – as are a successful labour outcome and non-medical factors such as cost, perceived quality of care and proximity to services. These components of care were influential on women's expectations which in turn influence acceptability and uptake of services. The recommendations emerging from this study reinforce the importance of provider awareness regarding attitude, and the need for development of inter-personal communication skills into education and training, alongside supporting supervisory mechanisms. Provider perceptions are also an important area to investigate to facilitate change in clinical practices. Recommended interventions should be supplemented with broader health systems improvements, including an understanding of the 'demand-inducing' factors which influence women's decision making for their delivery care.

## List of abbreviations

FGD Focus group discussion

FIGO International Federation of Gynecology and Obstetrics

GNP Gross national product

ICM International Confederation of Midwives

SAFE Skilled Attendance for Everyone

TBA Traditional birth attendant

WHO World Health Organization

## Competing interests

The author(s) declare that they have no competing interests.

## Authors' contributions

JH and MA conceived of the study, with the International SAFE research partnership. JH participated in the study design and co-ordination, contributed to the data analysis and helped to draft the manuscript. MA carried out the FGDs and in-depth interviews, conducted the data analysis and drafted the manuscript. LD contributed to the data analysis and led the drafting of the manuscript. All authors read and approved the final manuscript

## Pre-publication history

The pre-publication history for this paper can be accessed here:


